# Chickens, more than humans, focus the diversity of their immunoglobulin genes on the complementarity-determining region but utilise amino acids, indicative of a more cross-reactive antibody repertoire

**DOI:** 10.3389/fimmu.2022.837246

**Published:** 2022-12-08

**Authors:** Jessica Mallaby, Joseph Ng, Alex Stewart, Emma Sinclair, Deborah Dunn-Walters, Uri Hershberg

**Affiliations:** ^1^ Department of Bioscience and Medicine, University of Surrey, Guildford, United Kingdom; ^2^ Randall Centre for Cell and Molecular Biophysics, King’s College London, London, United Kingdom; ^3^ Department of Human Biology, University of Haifa, Haifa, Israel

**Keywords:** immunoglobulin, B cell, diversity, avian, human, amino acid

## Abstract

The mechanisms of B-cell diversification differ greatly between aves and mammals, but both produce B cells and antibodies capable of supporting an effective immune response. To see how differences in the generation of diversity might affect overall repertoire diversity, we have compared the diversity characteristics of immunoglobulin genes from domestic chickens to those from humans. Both use V(D)J gene rearrangement and somatic hypermutation, but only chickens use somatic gene conversion. A range of diversity analysis tools were used to investigate multiple aspects of amino acid diversity at both the germline and repertoire levels. The effect of differing amino acid usages on antibody characteristics was assessed. At both the germline and repertoire levels, chickens exhibited lower amino acid diversity in comparison to the human immunoglobulin genes, especially outside of the complementarity-determining region (CDR). Chickens were also found to possess much larger and more hydrophilic CDR3s with a higher predicted protein binding potential, suggesting that the antigen-binding site in chicken antibodies is more flexible and more polyreactive than that seen in human antibodies.

## Introduction

The adaptive immune systems of aves and mammals differ greatly, particularly in the development of B cells since aves possess a unique organ, the bursa of Fabricius. The bursa is the initial site of B-cell development prior to its involution of the bursa at 12 weeks of age (1). Avian species also rely on gene conversion (2) to generate diversity in their naïve repertoire. By contrast, in mammals, the bone marrow is the location of B-cell development, and diversity is created by gene rearrangement of a large number of possible V, D, or J genes (3). Comparing avian repertoires to human antibody repertoires provides insights into how naïve B-cell diversity can be generated using two different mechanisms.

In domestic chickens (*Gallus domesticus*) and humans (*Homo sapiens*), B cells start life as haemopoietic stem cells in the bone marrow. VDJ gene rearrangement creates immunoglobulin genes by bringing together a variable gene segment, a diversity gene segment–heavy chain only, and a joining gene segment. Humans possess a large selection of each gene segment, in contrast to chickens where only a single functional variable and joining gene segment are available in both the heavy and light chains. In chickens, the antibody heavy and light chains are rearranged simultaneously (4, 5), whereas in humans, the immunoglobulin light chain is rearranged first, initially using a kappa light chain. If this results in a non-functional immunoglobulin gene, then a second rearrangement is attempted using a lambda light chain (3). In both chickens and humans, the variable gene is subdivided into six regions: three framework regions (FWRs) and three complementarity-determining regions (CDRs). Following VDJ gene rearrangement in the chickens, B cells migrate into the bursa of Fabricius where they undergo further diversification by somatic gene conversion (SGC) and somatic hypermutation (SHM). SGC diversifies the immunoglobulin heavy and light chain variable genes through the insertion of genetic sequence from a library of non-functional variable genes (pseudogenes), which are located upstream of the functional variable gene [Davison. F, Avian Immunology]. SGC and SHM in chickens and SHM in humans are focused on the CDRs of the variable gene, as these regions form the antigen-binding site (6, 7).

Chicken monoclonal antibodies are currently used in a variety of immunotherapeutics. The inability of chicken antibodies to activate an immune response in humans and the ethical collection of these antibodies in large quantities from eggs make them ideal therapeutic candidates (8). An in-depth analysis of the molecular characteristics of avian antibodies is vital to enable the early identification of undesirable characteristics in the therapeutic development process. Previous research by Wu et al. highlighted key differences in the amino acid composition of chickens and humans (9). To further investigate the differences in amino acid diversity and identify how these differences relate to differences in other molecular and structural characteristics, we use next-generation sequencing (NGS) data to directly compare the B-cell antibody repertoire of chickens and humans, to further investigate the differences in amino acid diversity, and to identify how these differences relate to differences in other molecular and structural characteristics. We also determine how the use of different diversification mechanisms impacts the B-cell repertoire at the molecular level. Understanding how these antibodies differ could elucidate further areas in which avian immunoglobulins can be manipulated for use in therapeutics.

## Methods and materials

Chicken immunoglobulin sequences were sampled in six 3-week-old Rhode Island Red chickens from three immune-associated tissues: the bursa of Fabricius, caecal tonsil, and spleen. Samples were processed and sequenced as specified by Mallaby et al. (10). A total of 311,870 chicken heavy immunoglobulin sequences and 95,161 chicken lambda light chain immunoglobulin sequences were analysed. Human immunoglobulin sequences were generated from whole blood samples taken from 24 healthy donors; these samples were collected and processed as part of a study conducted by (11). Human donors ranged between the ages of 23 and 76. In total, 396,506 human heavy chain immunoglobulin sequences, 43,000 human kappa light chain sequences, and 158,668 human lambda light chain sequences were analysed. The genetic sequences of both human and chicken germline genes were downloaded from the International ImMunoGeneTics information system (IMGT) database. All sequences were processed using IMGT High V Quest and BRepertoire online analysis tools, which extracted the nucleotide and amino acid sequences of each FWR and CDR (12, 13).

### Comparison of germline and somatic diversity

Diversity scores were calculated for each amino acid position using diversity and richness metrics, developed by Schwartz and Hershberg (14). This calculated a diversity score with values in a range of 1–21 counting the number of different amino acids present at each nucleotide position. A diversity value of 21 could be assigned, as the two codon conformations of serine (TCN or AGY) were calculated separately. A mean of this value across all clones was taken for each individual sampled, which was then visualised in a heatmap using GraphPad Prism (version 9.1.0). Due to the high levels of intra-species variation in CDR3 length, diversity scores were calculated from chickens and humans using sequences with CDR3s of average length, thus limiting this investigation to the analysis of 17% and 12% of chicken and human heavy chain immunoglobulins respectively, and 39%, 34%, and 67% of chicken lambda, human lambda, and human kappa light chains, respectively. As a result of inter-species variation, the diversity score for each CDR3 position could not be directly compared between species. Therefore, these data were then presented using a violin plot instead of a heatmap to display the range of diversity scores seen at positions within the CDR3. The difference in variation was analysed statistically using a one-way ANOVA, including Bonferroni corrections, to mitigate against multiple comparisons.

### Analysis of diversity change

With the use of the diversity scores calculated in the previous analysis, the diversity score given at each amino acid position for germline genes was subtracted from the diversity score calculated for observed repertoire sequences. When analysing the heavy chain diversity change in the chickens, the pseudogene diversity was subtracted from each antibody class separately. In the human heavy chain data, the diversity of the germline functional genes was subtracted from the IgM repertoire diversity, and then the diversity of the IgM repertoire was subtracted from both secondary antibody classes (IgA and IgG) separately. The range of values was then statistically analysed using Wilcoxon rank sum test, analysing the difference from a theoretical difference of 0.

### Calculation of activation-induced deaminase hotspots in the variable region

The number of activation-induced deaminase (AID) hotspots in both RGY and WRC (AAC, AGC, TAC TGC, AGT, GGC, and GGT) conformations was counted (15–17). They were counted both in and out of the reading frame, as the AID enzyme is not restricted to reading codons in-frame. Hotspots were counted for the FWRs and CDRs separately in both species for the functional genes. They were also counted for the pseudogenes in chickens only. The numbers were converted to a percentage using the total number of codons in combined FWRs and CDRs. A percentage of AID hotspots were calculated for each gene sequence, and the Mann–Whitney test was used to analyse the significance of the results.

### Calculation of complementarity-determining region length

The total amino acid length was calculated for each CDR in the repertoire sequences of both humans and chickens, using the BRepertoire™ online analysis tool (13).

### Comprehensive analysis of amino acid usage in the complementarity-determining regions at the germline level

The variable region sequence of germline genes of functional and pseudogenes in chickens and the functional genes of humans at both the heavy and light chain loci were divided into the FWRs and CDRs. The occurrence of each amino acid was calculated for each sequence. Serine residues were divided into two types, serine 1 (Ser1) and serine 2 (Ser2), based on their codons, with TCN codons labelled as Ser1 and AGY codons labelled as Ser2. The number of each amino acid was converted to a percentage based on the total number of amino acid positions in all three FWRs and CDRs. These data were visualised on a dot plot with each dot representing an individual gene and a bar representing the average. One-way ANOVAs were then used to statistically analyse the difference between species.

### Analysing the usage of amino acids categorised by hydrophobicity

Amino acids were categorised by their hydrophobicity score. In this analysis, hydrophobic amino acids included phenylalanine (Phe), leucine (Leu), isoleucine (Ile), methionine (Met), valine (Val), cysteine (Cys), and tryptophan (Trp). Neutral amino acids included serine, proline (Pro), threonine (Thr), alanine (Ala), tyrosine (Tyr), and histidine (His). Hydrophilic amino acids included glycine (Gly), glutamine (Gln), asparagine (Asn), lysine (Lys), aspartic acid (Asp), glutamic acid (Glu), and arginine (Arg) (18, 19). They were analysed only in the germline CDRs. The occurrence of each amino acid from each category was totalled and converted to a percentage based on the total number of amino acids in the gene regions. The average was then visualised on a bar chart, and the data were statistically analysed using one-way ANOVA.

### Analysis of protein characterisation indices

Four indices were used to analyse different aspects of proteins—the aliphatic index, Boman index, hydrophobicity index, and instability index. BRepertoire™ online analysis tool (13) was used to calculate a value for each of the four indices for each genetic sequence.

The aliphatic index was calculated following the methods listed by Ikai (20). With the use of the mole percentage of aliphatic amino acids [alanine (*X_A_*), valine (X_V_), isoleucine (X_I_), and leucine (X_L_)], coefficients *a* and *b* were calculated as the relative volumes of aliphatic side chains to alanine side chains, following mathematical formula (21)


$$Aliphatic\;Index=x_{A}+2.9x_{V}+3.9\left(x_{I}+x_{L}\right)$$

The Boman index was calculated following methods listed by Boman (22). With the use of the following equation, the solubilities of each amino acid (**Supplementary Table 1**) are divided by the total sequence length (21):


$$Boman\;Index\;=\;\frac{\sum_{i=1}^{N}Si}{N}$$

The hydrophobicity index was calculated based on the amino acid hydrophobicity scores described by Kyte and Dolittle (23). This is determined by calculating the sequence total hydrophobicity by adding the hydrophobicity values described by Kyle and Dolittle (**Supplementary Table 2**) and dividing this value by the total number of amino acids using the following formula (21):


$$Hydrophobicity\;Index\;=\;\frac{\sum_{i=1}^{N}H_{i}}{N}$$

Finally, the instability index was calculated based on methods described by Guruprasad etal. (24). This index is calculated using the length of the sequence (L), *X*_*i*_ *Y*_*i*_ is the dipeptide value (**Supplementary Table 3**), and the dipeptide instability weight value (DIWV):


$$Instability\;Index=\;\frac{10}{L}\sum_{i=1}^{L}DIWV_{\left(X_{i}Y_{\left(i+1\right)}\right)}$$

These indices were analysed in both the heavy and light chain germline genes of both species; at the heavy chain loci, human pseudogenes were included in this analysis. At the repertoire level, heavy chain sequences were divided into different antibody classes, with comparisons drawn between human IgA and chicken IgA, human IgM and chicken IgM, and finally human IgG and chicken IgY. At the light loci, chicken lambda light chains were compared to both human lambda and human kappa light chains. The distribution of these values was analysed using the Mann–Whitney tests.

## Results

### Similarities and differences in germline and somatic diversity patterns between chickens and humans

**Figure 1:**
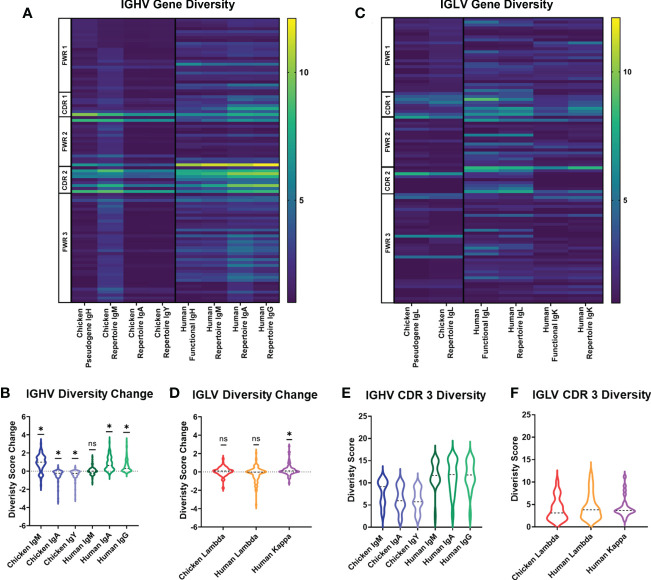
Diversity score analysis of each amino acid position in the germline immunoglobulin variable genes and repertoire sequences for each antibody class in both species. **(A)** Diversity score for each amino acid position of heavy chain germline and repertoire sequences. N: chicken heavy chain pseudogenes = 120, human heavy chain functional genes = 351, chicken IgA repertoire sequences = 7,273, chicken IgM repertoire sequences = 43,243, chicken IgY repertoire sequence = 47,871, human IgM repertoire sequences = 133,521, human IgA repertoire sequences = 166,524, and human IgG repertoire sequences = 96,113. **(B)** Analysis of diversity score change between germline and repertoire in heavy chain sequences of both chickens and humans. Negative diversity score change indicates a decrease in diversity, and positive diversity score change indicates increase in diversity. Statistical significance calculated using Wilcoxon rank sum test (WRST), analysing the difference between a theoretical mean of 0 and the actual median. p-Values: ns = p > 0.05, *p ≤ 0.05. **(C)** Diversity score for each amino acid position of light chain germline and repertoire sequences. N: chicken lambda light chain pseudogenes = 49, chicken lambda repertoire sequences = 38,244, human lambda light chain functional genes = 85, human kappa light chain functional genes = 99, human lambda repertoire sequences = 38,244, and human kappa repertoire sequences = 158,668. **(D)** Analysis of diversity score change between germline and repertoire in light chain sequences of both chickens and humans. Statistical significance calculated using WRST. p-Values: ns = p > 0.05, *p ≤ 0.05. **(E)** Analysis of diversity scores for amino acid positions in the CDR3 region of heavy chain antibody repertoires in both chickens (IgM, IgA, and IgY) and humans (IgM, IgA, and IgG). Statistical analysis was performed using a one-way ANOVA to compare the means using Bonferroni’s corrections to mitigate multiple comparisons. p-Values: ns = p > 0.05, *p ≤ 0.05. **(F)** Analysis of diversity scores for amino acid positions in the CDR3 region of light chain antibody repertoires in both chicken lambda and human lambda and kappa. Statistical analysis was performed using a one-way ANOVA to compare the means.

In order to assess variation in chicken and human immunoglobulin diversity, we compared heavy and light chain sequences between the germline genes contributing to naïve B-cell diversity, i.e., chicken germline pseudogenes and human germline functional genes. We also compared the diversity of different immunoglobulin heavy chain classes and light chain repertoires between chickens and humans. At the germline level, chicken pseudogenes have fewer amino acid positions showing high levels of diversity in comparison to the human germline functional genes (**Figure 1A**). Both species share the same diversity of hotspots, with the border of CDR1 and all of CDR2 being foci for amino acid diversity. In humans, the heavy chain repertoire is shown to be more diverse across the full length of the variable gene in all heavy chain classes. Observed repertoire sequences were expected to have a higher level of diversity when compared to the germline, due to somatic hypermutation events, and this was seen in human IgA and IgG repertoire, showing higher diversity scores at the majority of amino acid positions. In the chicken heavy chain, the IgM repertoire shows an increase in diversity. However, the majority of positions in IgA and IgY antibodies decrease in diversity (**Figure 1B**). In light chains, chicken lambda pseudogenes show a similar level of diversity to human kappa light chain functional genes with similar foci of diversity. Human lambda light chain functional genes differ from this, having diversity at more amino acid positions in comparison to chicken pseudogenes and human kappa (**Figure 1C**). Analysis of the change in diversity from germline to repertoire shows a similar distribution of amino acid positions that increase in diversity in both the chicken lambda pseudogenes and human kappa light chains (**Figure 1D**).

Due to variation in the CDR3 length between chickens and humans, the diversity score for each amino acid position in the CDR3 was displayed separately. In the heavy chain, chicken immunoglobulin CDR3 regions were found to be significantly less diverse than the CDR3 regions of human immunoglobulin genes in all antibody classes (**Figure 1E**), whereas in the light chain immunoglobulin repertoires, the CDR3 regions of all immunoglobulin classes in both species showed highly similar levels of amino acid diversity (**Figure 1F**).

### Activation-induced deaminase hotspot distribution

**Figure 2:**
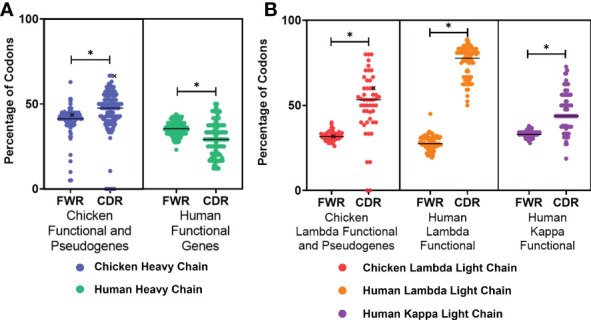
Proportion of activation-induced deaminase (AID) hotspots in the framework regions (FWR) and complementarity-determining regions (CDR), comparing **(A)** chicken heavy chain functional variable gene and variable pseudogenes against human functional variable genes and **(B)** chicken lambda light chain functional and pseudogene variable genes against human lambda functional genes as well as human kappa functional genes. Both RGY and WRC hotspots were included in the analysis (AAC, AGC, TAC, TGC, AGT, GGC, and GGT). Black “X” denotes the single chicken functional gene. Datasets were statistically analysed using a Mann–Whitney test. p-Values: *p ≤ 0.05.

The presence of AID hotspots is linked to the genetic diversification of immunoglobulin genes by somatic hypermutation in both species; therefore, the occurrence of these hotspots in the FWRs and CDRs of both species was analysed. In the heavy chain (**Figure 2A**), chickens were found to have a higher percentage of AID hotspots in both the FWRs and CDRs in comparison to the human functional variable genes; this difference was found to be statistically significant in the CDRs. The chicken functional variable gene (IGHV1-1*01) was found to have the highest proportion of AID hotspots in the CDRs. All light chain classes were found to have a significantly higher percentage of AID hotspots in the CDRs (**Figure 2B**). Chicken lambda light chains were found to be similar to the human kappa functional genes in regard to the distribution of AID targets, with human lambda light chains showing a greatly increased percentage of AID hotspots in the CDRs.

### Comparison of complementarity-determining region length

**Figure 3:**
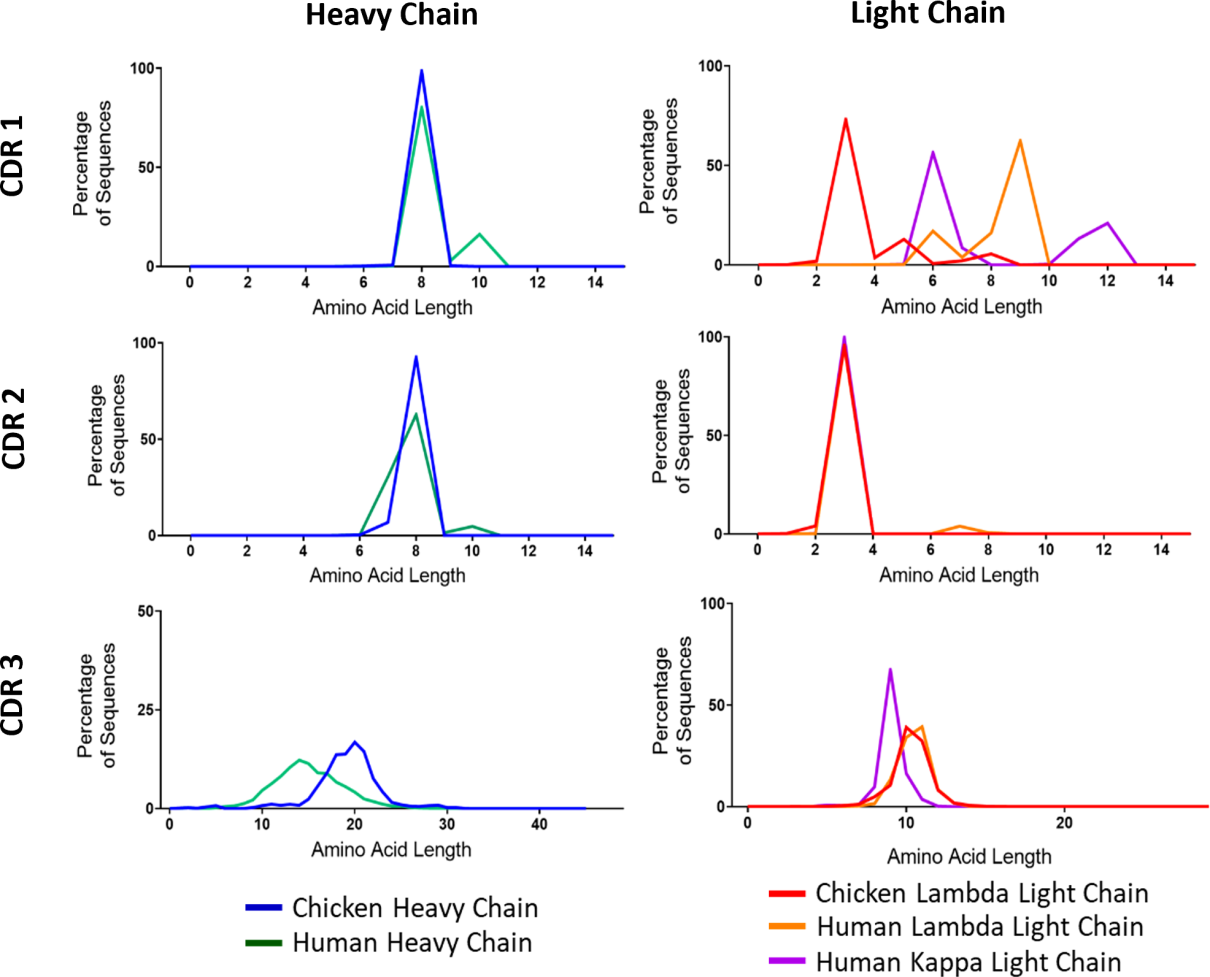
Distribution of amino acid lengths in all three complementarity-determining regions (CDRs) of both the immunoglobulin heavy and light chains. N: human heavy chain sequences = 396,506, chicken heavy chain sequences = 98,387, chicken lambda light chain sequences = 38,244, human lambda light chain sequences = 43,000, and human kappa light chain sequences = 158,668.

A major source of variability in heavy and light chains stems from variations in CDR3 length. The other CDRs also show some differences in length between V gene families. As a final comparison of diversity, we wanted to test to what extent these regions differed between chickens and humans. We found large amounts of variation between species in the CDR1 of light chain immunoglobulin repertoire sequences (**Figure 3**). Chickens have much smaller CDR1s, averaging at only three amino acids in length, whereas the human lambda chain had an average length of nine amino acids. In CDR3, light chain sequences between the species are very similar (**Figure 3**). There is a large interspecies variation in CDR3 length of heavy chains, with chickens displaying a higher mean length of 20 amino acids compared to 14 amino acids in humans (**Figure 3**).

### Differences in amino acid usage of chickens and humans at the germline level

**Figure 4:**
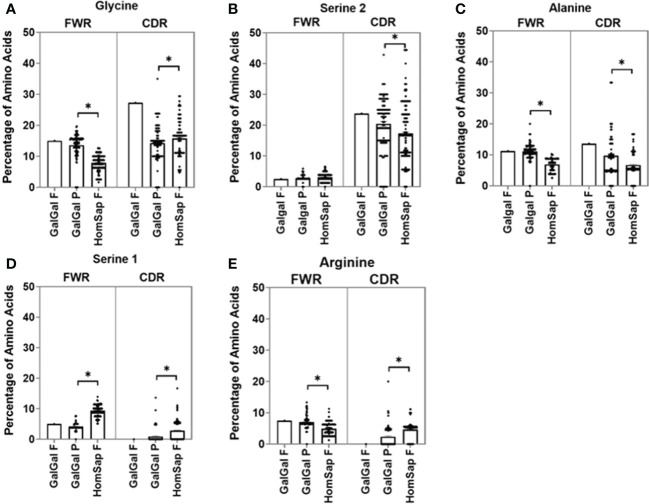
Comparison of amino acid usage across all three complementarity-determining regions (CDRs) of the heavy chain variable germline genes of both chickens and humans, displaying amino acid usage of **(A)** glycine, **(B)** serine 2 (AGY), **(C)** alanine, **(D)** serine 1 (TCN), and **(E)** Arginine. Datasets were statistically analysed using a one-way ANOVA. p-Values: *p ≤ 0.05.

We characterised and compared the amino acid usage in both the FWRs (**Supplementary Figure 1**) and CDRs (**Supplementary Figure 2**) of the variable gene. In this analysis, we focused on the CDRs since they form the antigen-binding site. We found significant differences in the usage of many amino acids. When investigating the heavy chain CDRs (**Supplementary Figure 2A**), the predominant amino acids in both species were found to be glycine (**Figure 4A**) and serine 2 (AGY) (**Figure 4B**). Chicken pseudogenes were found to have a significantly higher percentage of alanine (**Figure 4C**) residues and a significantly lower percentage of serine 1 (TCN) (**Figure 4D**) and arginine (**Figure 4E**) residues when compared to humans. In addition, no arginine residues were found in the single functional gene in the chickens.

**Figure 5:**
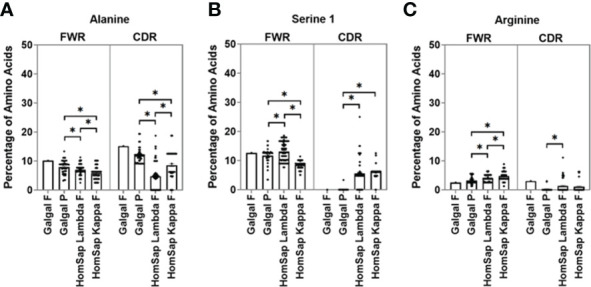
Comparison of amino acid usage across all three complementarity-determining regions (CDRs) of the light chain variable germline genes of both chickens and humans, displaying amino acid usage of **(A)** alanine, **(B)** serine 1 (TCN), and **(C)** arginine. Datasets were statistically analysed using a one-way ANOVA. p-Values: *p ≤ 0.05.

In the CDRs of heavy chain immunoglobulin genes, both species showed a greater variety of amino acids in comparison to the light chain (**Supplementary Figure 2B**). Alanine was found to be present in chicken pseudogenes at a significantly higher percentage than in humans (**Figure 5A**), and serine 1 (TCN) was found to be significantly lower in chickens (**Figure 5B**), with no serine 1 residues found in the single functional variable gene. The percentage of arginine residues in the chicken pseudogenes was found to be similar to that seen in human kappa light chains but significantly lower than that seen in the human lambda light chain (**Figure 5C**). A higher percentage of arginine residues were found in the chicken functional gene in comparison to that of human lambda and kappa light chains.

### Analysis of protein structure characteristics

We identified significant differences in amino acid sequence between the two species. The impact of these amino acid differences was investigated further by analysing a number of characteristics, which are affected by the amino acid sequence and, in turn, can affect the structure and shape of the resulting protein. These include the aliphatic index, hydrophobicity, Boman index, and instability index, all of which were calculated using the BRepertoire™ online data analysis tool (see Methods and Materials).

#### Analysis of the aliphatic properties of the germline and repertoire genetic sequences

**Figure 6:**
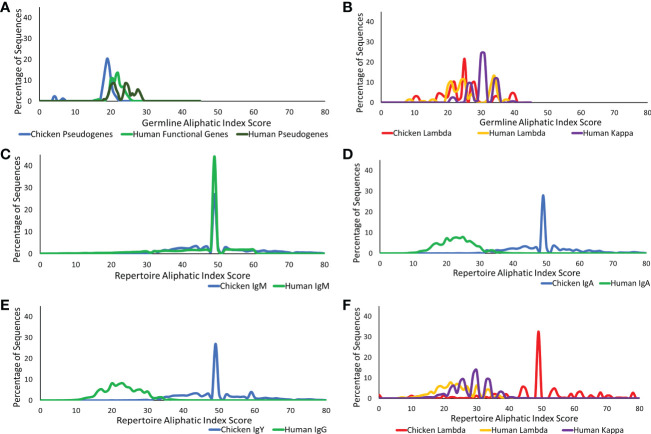
Aliphatic index comparison of germline variable genes between **(A)** chicken pseudogenes, human functional genes, and human pseudogenes at the heavy chain loci and **(B)** chicken pseudogenes, human lambda functional genes, and human kappa functional genes at the light chain loci. Comparison of the aliphatic index between repertoire sequences shows **(C)** chicken IgM against human IgM, **(D)** chicken IgA against human IgA, **(E)** chicken IgY against human IgG, and **(F)** chicken lambda light chain sequences against human lambda light chain sequences and human kappa light chain sequences. Aliphatic index score calculated by BRepertoire online immunoglobulin repertoire analysis tool using the CDR3 region amino acid sequence.

The presence of aliphatic side chains in globular proteins impacts the thermostability of the protein. The aliphatic index calculates the volume of aliphatic side chains that are composed of alanine, isoleucine, leucine, and valine amino acids to estimate the thermostability of the protein (20, 25). This was calculated for the CDR3 region germline genes and repertoire sequences in both the heavy and light chains. At the germline level of the heavy chain loci in chickens and humans, chickens were found to have a significantly lower volume of aliphatic side chains (**Figure 6A**), whereas in the light chain, chickens were found to have a similar aliphatic index in comparison to human functional genes (**Figure 6B**).

The aliphatic index of heavy chain repertoire sequences in humans and chickens was analysed in each antibody class separately (**Figures 6C–E**). At the repertoire level, IgM antibodies in both species were found to be highly similar (**Figure 6C**); however, the human class-switched IgA (**Figure 6D**) and IgG (**Figure 6E**) show a dramatic reduction in the presence of aliphatic side chains. In chickens, an increase in the aliphatic index is seen between germline and repertoire (**Figure 6**). A comparison of antibody classes in the chickens identified that the aliphatic index distribution remains consistent across all antibody classes (**Figures 6D, E**). Chicken lambda light chains at the repertoire level show a significantly higher aliphatic index, in comparison to both human lambda and kappa (**Figure 6F**).

#### Variation in hydrophobic amino acid usage in complementarity-determining regions of variable germline genes

**Figure 7:**
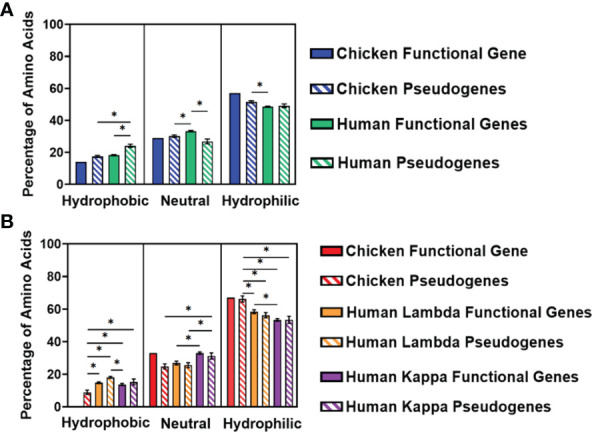
Comparison of amino acid usage across all three complementarity-determining regions (CDRs) of the variable germline genes of both chickens and humans, categorising amino acids by their hydrophobicity, displaying amino acid usage in **(A)** the immunoglobulin heavy chain and **(B)** immunoglobulin light chain. Hydrophobic amino acids, phenylalanine, (Phe); leucine, (Leu); isoleucine, (Ile); methionine, (Met); valine, (Val); cysteine, (Cys); and tryptophan, (Trp); Neutral amino acids, serine; proline, (Pro); threonine, (Thr); alanine, (Ala); tyrosine, (Tyr); and histidine, (His); Hydrophilic amino acids, glycine, (Gly); glutamine, (Gln); asparagine, (Asn); lysine, (Lys); aspartic acid, (Asp); glutamic acid, (Glu); and arginine, (Arg). Datasets were statistically analysed using a one-way ANOVA. p-Values: *p ≤ 0.05.

We separated the amino acids into three categories—hydrophobic, neutral, or hydrophilic—following the categorisation described by Schwartz etal. (18). The percentage of amino acids in each category was calculated for the three CDRs and displayed as an average (**Figure 7**). In the heavy chain, there is a preference for hydrophilic amino acids in both chickens and humans, with chicken pseudogenes showing a significantly higher percentage of hydrophilic amino acids compared to human functional genes (**Figure 7A**). Similarly, hydrophilic amino acids were preferred by both species in the light chain, particularly in chickens, which showed a significantly higher percentage of hydrophilic amino acids in comparison with both human lambda and kappa light chains (**Figure 7B**).

**Figure 8:**
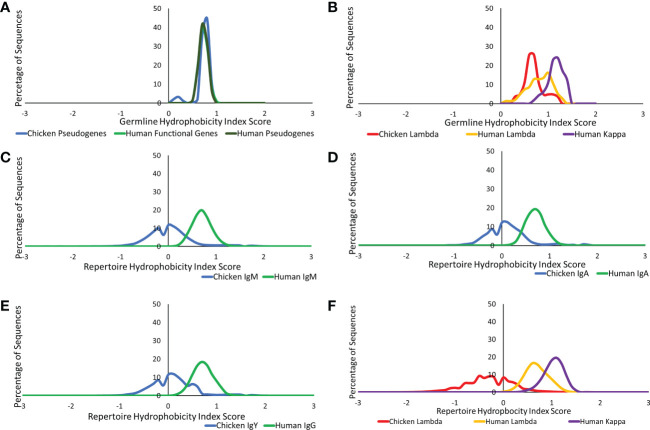
Comparison of hydrophobicity of germline variable genes between **(A)** chicken pseudogenes, human functional genes, and human pseudogenes at the heavy chain loci and **(B)** chicken pseudogenes, human lambda functional genes, and human kappa functional genes at the light chain loci. Comparison of the hydrophobicity between repertoire sequences shows **(C)** chicken IgM against human IgM, **(D)** chicken IgA against human IgA, **(E)** chicken IgY against human IgG, and **(F)** chicken lambda light chain sequences against human lambda light chain sequences and human kappa light chain sequences. Hydrophobicity index score calculated by BRepertoire online immunoglobulin repertoire analysis tool using the CDR3 region amino acid sequence.

We further investigated the difference in amino acid usage *via* the calculation of the hydrophobicity index (23) in the CDR3 of both species at the germline and repertoire levels. Chickens and humans were found to have highly similar hydrophobicity scores in the heavy chain germline genes (**Figure 8A**), but chicken light chains were less hydrophobic than human light chains (**Figure 8B**). At the repertoire level in the heavy chain (**Figures 8C–E**), chickens were found to be significantly less hydrophobic in all antibody classes. A decrease in hydrophobicity was seen in chicken repertoire sequences compared to the chicken germline gene. The average hydrophobicity in class-switched human heavy chains remains consistent with that seen at the germline level, although the distribution range increased in all antibody classes (**Figures 8C–E**). The hydrophobicity of chicken light chain repertoire sequences is significantly lower than that seen in the human lambda and kappa light chains and differs greatly from the distribution seen at the germline level (**Figure 8F**). The hydrophobicity of both the human light chains remains consistent with that seen at the germline level.

#### Analysis of the protein binding potential of the germline and repertoire genetic sequences

**Figure 9:**
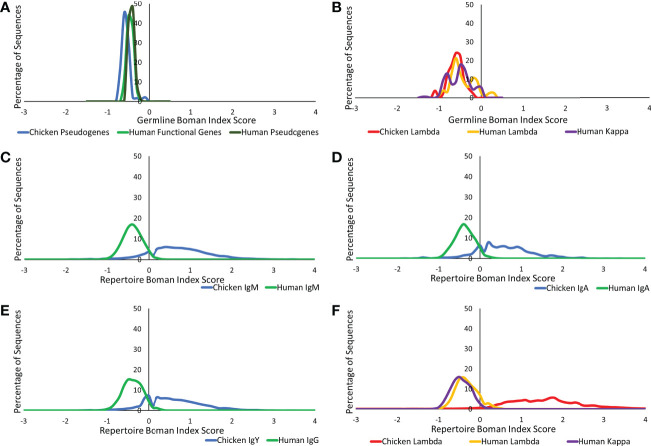
Protein binding potential (Boman index) comparison of germline variable genes between **(A)** chicken pseudogenes, human functional genes, and human pseudogenes at the heavy chain loci and **(B)** chicken pseudogenes, human lambda functional genes, and human kappa functional genes at the light chain loci. Comparison of the protein binding potential between repertoire sequences shows **(C)** chicken IgM against human IgM, **(D)** chicken IgA against human IgA, **(E)** chicken IgY against human IgG, and **(F)** chicken lambda light chain sequences against human lambda light chain sequences and human kappa light chain sequences. Boman index score calculated by BRepertoire online immunoglobulin repertoire analysis tool using the CDR3 region amino acid sequence.

The Boman index is an estimation of a protein binding potential and can be used to assess the ability of a protein to bind to a variety of proteins (22, 26), a particularly important characteristic in antibody–antigen binding. At the germline level, similarities were found between chickens and humans in both the heavy and light chains (**Figures 9A, B**). In the observed repertoire, all heavy chain antibody classes show a higher protein binding potential in the chickens than in humans, although the distribution is less Gaussian in chickens (**Figures 9C–E**). When comparing the germline and repertoire values, human repertoire sequences have a Boman index distribution, which is consistent with that of the germline functional genes. Conversely, in chickens, there is a dramatic shift in the distribution from that seen in the germline. The lambda and kappa light chain repertoire sequences in humans show a Boman index distribution that is consistent with what is seen at the germline level; however, the distribution is more defined with less variability at the repertoire level (**Figure 9F**). At the repertoire level, the chicken lambda light chain shows a significantly higher and a broader range of protein binding potential in comparison to the human repertoire and chicken germline.

#### Analysis of the protein instability index of the germline and repertoire genetic sequences

**Figure 10:**
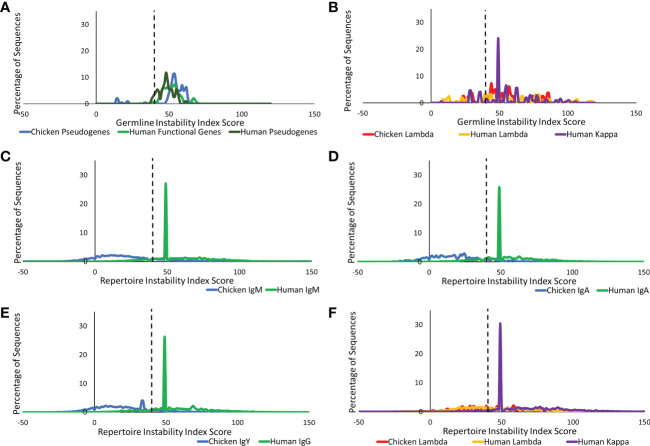
Comparison of protein instability of germline variable genes between **(A)** chicken pseudogenes, human functional genes, and human pseudogenes at the heavy chain loci and **(B)** chicken pseudogenes, human lambda functional genes, and human kappa functional genes at the light chain loci. Comparison of the protein binding potential between repertoire sequences shows **(C)** chicken IgM against human IgM, **(D)** chicken IgA against human IgA, **(E)** chicken IgY against human IgG, and **(F)** chicken lambda light chain sequences against human lambda light chain sequences and human kappa light chain sequences. Protein instability index score calculated by BRepertoire online immunoglobulin repertoire analysis tool using the CDR3 region amino acid sequence.

The instability index uses the presence of particular dipeptides within a protein to estimate the protein’s stability (24). A sequence with a value greater than 40 is described as an unstable protein (27). Large amounts of variation were seen at the germline level in the heavy and light chains of both species, with over 90% and over 60% of values falling above 40 in the heavy and light chains, respectively (**Figures 10A, B**). At the repertoire level in heavy chain sequences, human antibody classes showed a consistent highly specific distribution around a value of 50 (**Figures 10C–E**), which is an increase in instability from that seen at the germline level. In contrast, all chicken antibody classes had a broader range of index scores with the majority of values falling below the 40 thresholds for stability (**Figures 10C–E**). In the IgY class of antibodies, there is a multimodal distribution containing two peaks with index scores of 40 and 50 (**Figure 10E**). At the repertoire level in the light chain (**Figure 10F**), there is a reduction in variability in both species, with all light chains showing highly specific distributions, with the majority of sequences generating values of 50.

In summary, analysis of these four characteristics identified clear differences between the CDR3 regions of chickens and humans. We found that at the repertoire level across both the heavy and light chains, chickens had a higher occurrence of aliphatic side chains, are less hydrophobic, have a higher protein binding potential, and are more stable in experimental conditions in comparison to humans.

## Discussion

In the above study, we have determined that the diversity generated in the immunoglobulin variable gene of chickens is dramatically different from that of humans at the germline level and during the formation of repertoire diversity. Previous analysis of chicken repertoires, while limited, resulted in observations corroborated by our findings. However, a more comprehensive analysis was necessary to fully understand how chickens and humans differ in their repertoire diversity and makeup. At the germline level, chickens displayed a much more limited pattern of diversification that focused on fewer sequence positions than those found to be diverse in the human germline genes. Diversity was seen to increase from the germline level to the repertoire level as expected in humans (**Figure 1B**). The additional increase in diversity seen between IgM and secondary antibodies IgA and IgG could be a result of additional rounds of diversification *via* class-switched recombination and SHM following B-cell activation after exposure to immunogenic antigens (28, 29). In contrast, in chickens, we observed a reduction in diversity at the repertoire level in comparison to that seen at the germline level (**Figure 1**). This could potentially be a result of the skewed and preferential selection of pseudogenes during SGC identified in previous studies by (10). Previous investigations have also suggested that there is an evolutionary advantage to possessing a diverse range of variable immunoglobulin genes and that pseudogenes are less affected by this evolutionary drive and therefore are less diverse (30). Comparison of variable pseudogene diversity in the chicken and functional variable gene diversity in the human in this study supports this hypothesis.

In both species, the enzyme AID plays an important role in aiding the diversification of variable genes through either gene conversion or somatic hypermutation. Chicken heavy chain immunoglobulin pseudogenes and functional genes were found to have a higher proportion of AID hotspots in both FWRs and CDRs (**Figure 2A**). This higher proportion of hotspots in chicken heavy chains is likely the result of their reliance on AID-dependent gene conversion to introduce variability, whereas humans rely on RAG1/RAG2 dependent V(D)J recombination from a more diverse pool of functional genes. Comparatively, the chicken lambda light chain contains more AID hotspots than the heavy chain, a similar pattern to that seen in human kappa light chains (**Figure 2B**). The difference between heavy and light chains is likely the result of the limited diversification options available to chickens, only this time compounded by the lack of diversity genes, meaning they are reliant only on gene conversion for diversity. It is also worth noting that, as with the heavy chain, the lower level of AID hotspots in human kappa compared to chicken lambda is the result of multiple functional variables and joining genes and so a lower reliance on other mechanisms for diversification. The difference between human and chicken light chains may be the result of the developmental inclusion of chicken lambda and human kappa first, with human lambda being included only if kappa fails to create a functional gene (31, 32). The higher number of AID hotspots in human lambda may therefore compensate for a smaller pool of functional genes and reflect the importance of human lambda genes as the last resort to rescue a functional immunoglobulin, both of which could lead to a greater need to induce diversity through other mechanisms. The location of the hotspots also plays a key role in the conservation and diversification of specific areas, the higher proportion of AID hotspots in the CDRs allows for diversification to be focused on the antigen-binding site, and the FWRs are conserved to aid the maintenance of the proteins’ 3D structure. The fact that we also find AID targets in chicken pseudogenes suggests that mutations are also targeted to them when they are recombined into the variable gene.

Our comparison of the amino acid length of the CDRs in both the heavy and light chains of both species identified differences in the length of CDR1 in the light chain and the CDR3s of both the heavy and light chains (**Figure 3**). Chickens displayed a significantly longer CDR3 length in the heavy chain, whereas the CDR3 chicken lambda light chains were found to be similar to the human lambda light chain and longer than the kappa light chain. The CDR1 regions of light chains in chickens were found to be shorter than those of human lambda and kappa light chains. The light chain CDR1 region is believed to have more of an impact on the shape of the antigen-binding site in comparison to the heavy chain CDR1 region. Reducing the amino acid length of this region could therefore allow for better conservation of the overall shape of the antigen-binding site. Longer light chain CDR1 regions have been found to improve the binding affinity of antibodies to peptide antigens, suggesting that human antibodies may be better adapted to binding smaller peptide antigens, while chickens are better adapted to binding larger full-length proteins (33–35). The reduction in CDR1 length in chickens could also be in part compensation for the increased length of the heavy chain CDR3 because in the 3D structure of the antigen-binding site, the CDR1 hypervariable loop is located in close proximity to CDR3 loops of both the heavy and light chains (36). Previous research found the upper limits of chicken and human CDR3s to be 32 and 37, respectively (9), although the majority (89%) fall within the range identified in this study.

Differences in the amino acid usage between the two species can give key insights into different factors that impact antigen binding and protein structure. In this study, the two serine codons were analysed separately due to differences in their role of gene diversification, as serine 1 is an AID cold spot in comparison to serine 2, which is an AID hotspot. As has long been observed in humans (37), serine 1 (TCN) was found to occur more within the FWRs in both species when compared to serine 2 (AGY) (**Supplementary Figure 1**). Conversely, in the CDRs, serine 2 was found at a higher proportion than serine 1 (**Supplementary Figure 2**). Serine 1 codon conformations are a single mutation away from a stop codon, so AID will have been under evolutionary pressure not to target TCN for mutation, and these codons are mutated at a reduced rate to prevent the occurrence of a stop codon in the framework region. Serine 1 was found at a lower proportion in chicken FWRs and CDRs in comparison to human FWRs and CDRs, suggesting evolution away from the potential for acquiring stop codons (**Supplementary Figures 1**, **2**) (18). Serine 2 (AGY) is a more suitable candidate for the hypervariable CDRs because both codon variations of this amino acid are targets for the AID enzyme. and there is no risk of stop codon generation. The occurrence of serine 2 residues in the FWRs of both species was found to be similar; however, chickens were found to have a significantly higher proportion of these residues in the CDRs (**Supplementary Figures 1**, **2**). This could be due to the stronger reliance of chickens on AID-dependent diversification processes.

Small differences in the amino acid composition of proteins can result in drastic differences in the structural, functional, and physiochemical properties of the proteins. Therefore, the amino acid composition of chickens and humans was compared. In both species, the dominant amino acid residues in the variable region CDRs were found to be glycine, serine 2 (AGY), and alanine. However, all three residues were found to be present at significantly higher proportions in the CDRs of chickens (**Supplementary Figure 2**). Previous research suggests that the increased proportion of these amino acids is a result of the incorporation of IGHD genes that are rich in these amino acids (9). It has also been suggested that these amino acids, particularly glycine and serine 2, can induce sharp turns into a protein 3D structure (38). The inclusion of these sharp turns means that chicken CDRs could create antibodies with antigen-binding sites that are more dynamic in shape in comparison to human antibodies. The inclusion of alanine has also been suggested to influence the flexibility of the antigen-binding site (39). Therefore, simply varying the quantity and location of these residues within the antigen-binding site could potentially create an antibody repertoire with a diverse range of antigen-binding site conformations. The incorporation of cysteine residues in the CDRs of avian immunoglobulin genes has been suggested to cause the formation of disulfide bridges between CDR loops in the antigen-binding site, the presence of which can improve the stability of the resulting protein structure (9). The identification of significantly higher proportions of cysteine residues in the CDRs of chicken immunoglobulins in comparison to humans (**Supplementary Figure 2**) alongside the increased protein stability identified by the calculation of the instability index (**Figure 10**) supports the hypothesis that these residues may generate interdomain disulfide bonds, which influence protein stability.

The presence of arginine was also analysed due to its high charge resulting in DNA binding capabilities that have been associated with autoreactive potential (40–42). Chicken pseudogenes had significantly lower proportions of arginine residues in comparison to humans in both the heavy and light chains, with no arginine residues found in the heavy chain functional gene. The exclusion of arginine residues may be advantageous, as this would reduce the likelihood of developing antibodies that are capable of binding DNA and causing autoimmune disorders. The exclusion of arginine may balance the effects of more broadly reactive chicken antibodies, which would have a higher likelihood of being autoreactive.

The high abundance of aliphatic side chains in IgM antibodies of both species suggests that these antibodies are thermostable proteins. This high level of thermostability remains in all antibody classes in chickens but decreases in human secondary antibodies (25), suggesting that during an immune response in humans, there is a selection preference for antibodies with lower thermostability to undergo class switching. This could also suggest that a higher thermostability and a higher presence of aliphatic side chains are beneficial in antibodies with a specific effector function.

Hydrophobicity is a key factor in the 3D structure and the antigen-binding ability of an antibody, as it provides a strong attracting force within DNA molecules. Antigen-binding sites that are more hydrophobic are more capable of capturing antigens from further away (43). In this study, the antibodies of humans were found to be more hydrophobic (**Figures 7**, **8**), therefore suggesting that human antibodies are capable of capturing antigens from longer distances in comparison to chicken antibodies. In addition, hydrophobic antigen-binding sites are more likely to be located within the protein’s core, which in turn reduces the flexibility of the antigen-binding site (44), suggesting that chickens are potentially more flexible in comparison to human antibodies. This also supports the idea that chicken antibody-binding sites are more likely to have protruding structures caused by longer heavy chain CDR3s, which would be difficult to contain within the protein core.

The Boman index of both the heavy and light chain chicken repertoire sequences was higher and more broadly distributed than that of the human repertoire, suggesting that the avian repertoire has a larger variety of binding specificities than that of humans. The higher protein binding potential of chicken antibodies suggests that they may be able to form more broadly reactive antibodies that interact with a wider range of antigens in comparison to human antibodies (26). The broader distribution of values calculated for chicken immunoglobulins suggests the presence of an antibody repertoire with a diverse range of binding potentials. In comparison, the narrower ranges of human heavy and light chains suggest that all antibodies produced have very similar protein binding potentials.

The instability index estimates the stability of a protein in solution, and a broad range of index scores were found in both species. The higher proportion of index scores under 40 in chickens suggests that they potentially produce antibodies that are more stable in experimental conditions when compared to human antibodies (27). IgY in chickens had peak instability values just below a value of 40, suggesting that out of the three classes identified in chickens, IgY is potentially the most stable. This analysis is limited to the stability of a protein in experimental conditions, as the stability of a protein is not only limited to the amino acid structure of the protein but also impacted by the environment around it (45). Despite this, it goes hand in hand with our finding on the aliphatic index that the chicken antibodies are more stable and cross-reactive.

Genetic analysis of polyreactive antibodies has identified four characteristics that can influence the promiscuity of an antibody. While they have not been proven to be definitive characteristics, they can still be helpful indicators for identifying antibodies that have the potential to be polyreactive. The four factors with influence over polyreactivity are 1) heavy chain variable gene diversity, 2) heavy chain CDR3 length, 3) hydrophobicity of heavy chain CDR3, and 4) the presence of isoleucine residues in the CDR3 (46–50).

We found several differences in amino acid sequence makeup that could increase polyreactivity in chickens. Previous investigations into the polyreactivity of antibodies have identified the heavy chain variable region gene as having the biggest influence on the ability of an antibody to bind multiple antigens, particularly the CDR3 region, as the heavy chain CDR3 is located in the centre of the antigen-binding site and contributes the largest number of residues that interact with antigens (46–49, 51, 52). Investigations into the impact of class switching on polyreactive antibodies showed that changes in the Fc region of the antibody had no impact on the polyreactivity of an antibody (53). Studies conducted by Ichiyoshi and Casali (47) showed that inserting the heavy chain CDR3 region of a polyreactive antibody into a monoreactive antibody resulted in the inducement of polyreactive binding, indicating that the variable region is solely responsible for determining the polyreactive capabilities of an antibody.

Several studies have identified correlations between IGHV immunoglobulin regions that contain fewer genetic mutations and the occurrence of antibody polyreactivity. This may be a result of but not limited to the increased flexibility of the antigen-binding site seen in unmutated IGHV gene regions (49, 52, 54). We found that chicken immunoglobulin genes were found to be less diverse, as a result of high genetic similarity between the single functional IGHV gene and the library of pseudogenes (**Figure 1**). While this does not necessarily indicate that all chicken immunoglobulins are polyreactive, it is possible that polyreactive immunoglobulins may be more likely to occur due to the restrictive nature of gene conversion as a tool for diversification.

Heavy chain CDR3 length has been identified as a key factor in polyreactive antibodies, as longer CDR3s have the potential to present a wider range of residues within the antigen-binding site, thus providing a wider range of antigen contact points and enabling binding of multiple antigens (46–50). However, investigations of CDR3 length in polyreactive antibodies have would not support this, as human polyreactive antibodies have a varied range of CDR3 lengths (47). With this in mind, CDR3 length alone may not be a reliable predictor of polyreactivity, but the presence of much larger CDR3s in the chickens (**Figure 3**) may provide more opportunities for the presentation of a wider variety of antigen contact points within the antigen-binding site.

The third potential predictor is the hydrophobicity of the heavy chain CDR3, with an antigen-binding site that is more hydrophilic, being found to correlate with polyreactivity. A less hydrophobic binding site would also result in a weaker binding potential, which is a key characteristic of polyreactive antibodies (49). Analysis of hydrophobicity in the CDR3 region found that the chicken CDR3s are less hydrophobic in all heavy chain antibody classes as well as the light chain classes (**Figure 8**), suggesting that all classes of chicken antibodies have a greater potential to be polyreactive in comparison to human antibodies.

Finally, the presence of isoleucine residues in the heavy chain CDR3 has been shown to correlate with a significant reduction in antibody polyreactivity (49). Investigation of amino acid usage in the CDRs of chicken and human immunoglobulins found significantly fewer isoleucine residues in avian CDRs (**Supplementary Figure 2A**), further suggesting that avian antibodies have the potential to be more polyreactive.

In summary, chicken antibody genes were shown to be less genetically diverse in comparison to human antibody genes, particularly in the heavy chain immunoglobulin variable gene. In the absence of a diverse library of variable genes, gene conversion is sufficient to generate an antibody repertoire capable of initiating an effective immune response despite causing limited amino acid diversity. However, amino acid diversity is not the only factor contributing to the success of an antibody. The longer less hydrophobic CDR3s of chicken antibodies alongside the higher protein binding potential suggest that chicken antibodies could be more flexible in their binding, thus increasing the potential for polyreactive antibodies. This could be a way for chickens to mitigate for lack of repertoire diversity during early life stages, but this would be disadvantageous when using chickens as a model species for the production of immunotherapeutic antibodies, as it would increase the risk of non-specific binding. Research conducted by Schusser et al. may provide a solution to this (55, 56). By incorporating human-derived variable genes into the functional and pseudogene loci of DT40 immunoglobulin genes, humanised antibodies that were diversified *via* gene conversion were generated. Replacing chicken variable genes with human variable genes could be beneficial to the production of suitable immunotherapeutic candidates, as it could reduce the risk of generating polyreactive antibodies.

A caveat of this study was that human samples were collected from individuals over the age of 18, whereas chicken samples were collected from samples that were only 3 weeks in age and were raised in a pathogen-free environment. The immunological experience of the human samples, whether a result of vaccination or infection, allowed for the sampling of B cells at different stages of differentiation from naïve to memory. The chickens that were sampled were still very young. Previous studies have indicated that antigens derived from the gut and cloaca are circulated through the bursa, resulting in the activation and differentiation of B cells within the bursal follicle (57, 58) and suggests that, despite sampling at such a young age, these samples may still have included B cells at different stages. The limited sampling and the age at which chickens were sampled were limited due to compliance with ethical guidance. As a result, further research investigating individuals of equivalent ages is recommended.

The conclusions of this study are limited to the analysis of sequence data. Further experimental analysis is required to test and compare the polyreactive binding capabilities of antibodies from both species. However, this research has identified key characteristics that should be assessed when considering chickens are model species for immunotherapeutics.

## Data availability statement

Publicly available datasets were analyzed in this study. This data can be found here: https://zenodo.org/record/5636475#.YbsapWjP1PY https://zenodo.org/record/5146019#.YbsbQmjP1PY.

## Author contributions

Experimental data were supplied by JM, AS, and ES. JM, JN, AS, and UH conducted the bioinformatics analysis. All authors contributed and approved the submitted version.
