# Supplementary figures and images for: Chickens, more than humans, focus the diversity of their immunoglobulin genes on the complementarity-determining region but utilise amino acids, indicative of a more cross-reactive antibody repertoire
Source: Front Immunol. 2022 Dec 8;13:837246. doi: 10.3389/fimmu.2022.837246 (PMC9772431; doi:10.3389/fimmu.2022.837246)

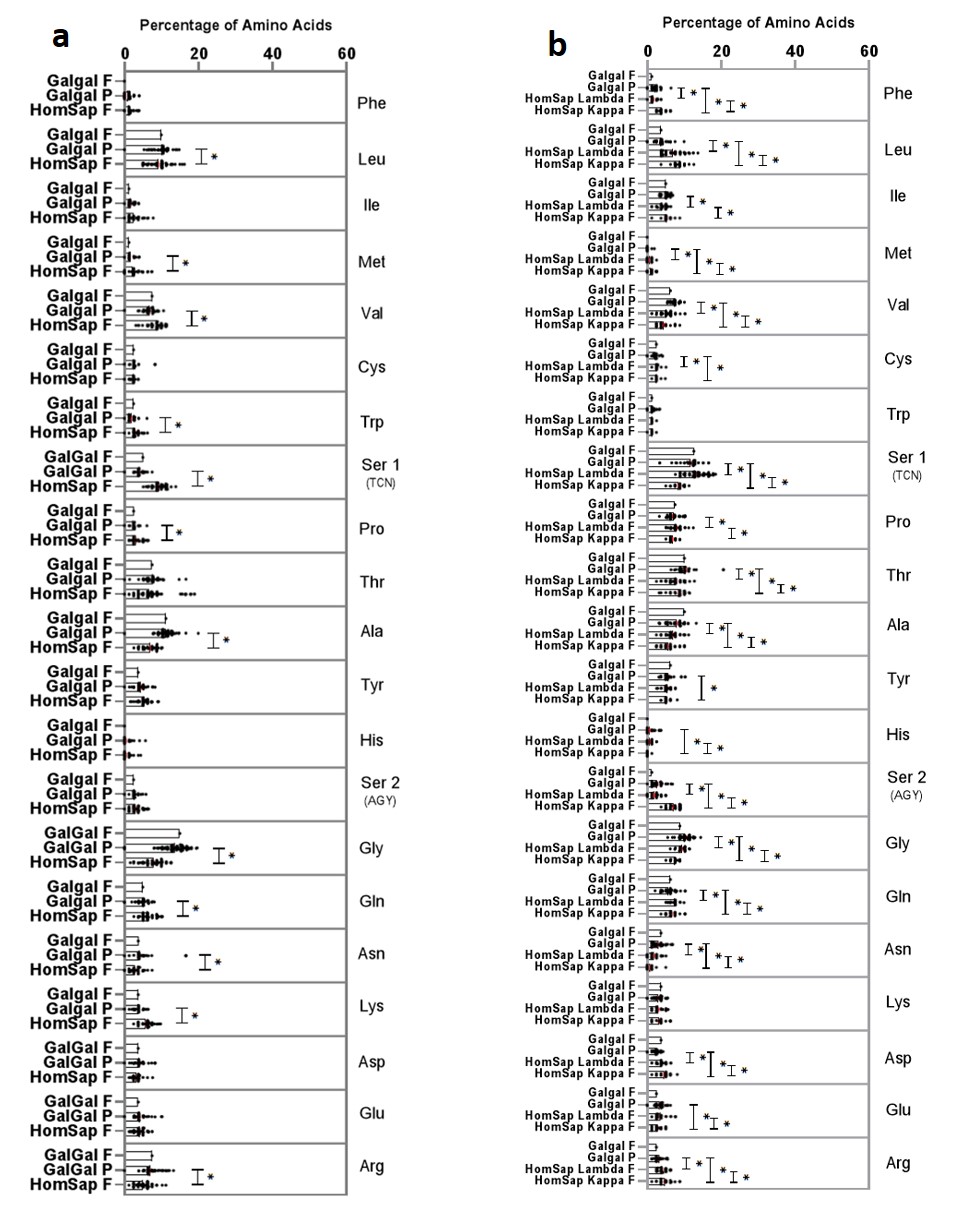

Supplement: Supplementary file 1 [file Image_1.jpeg]

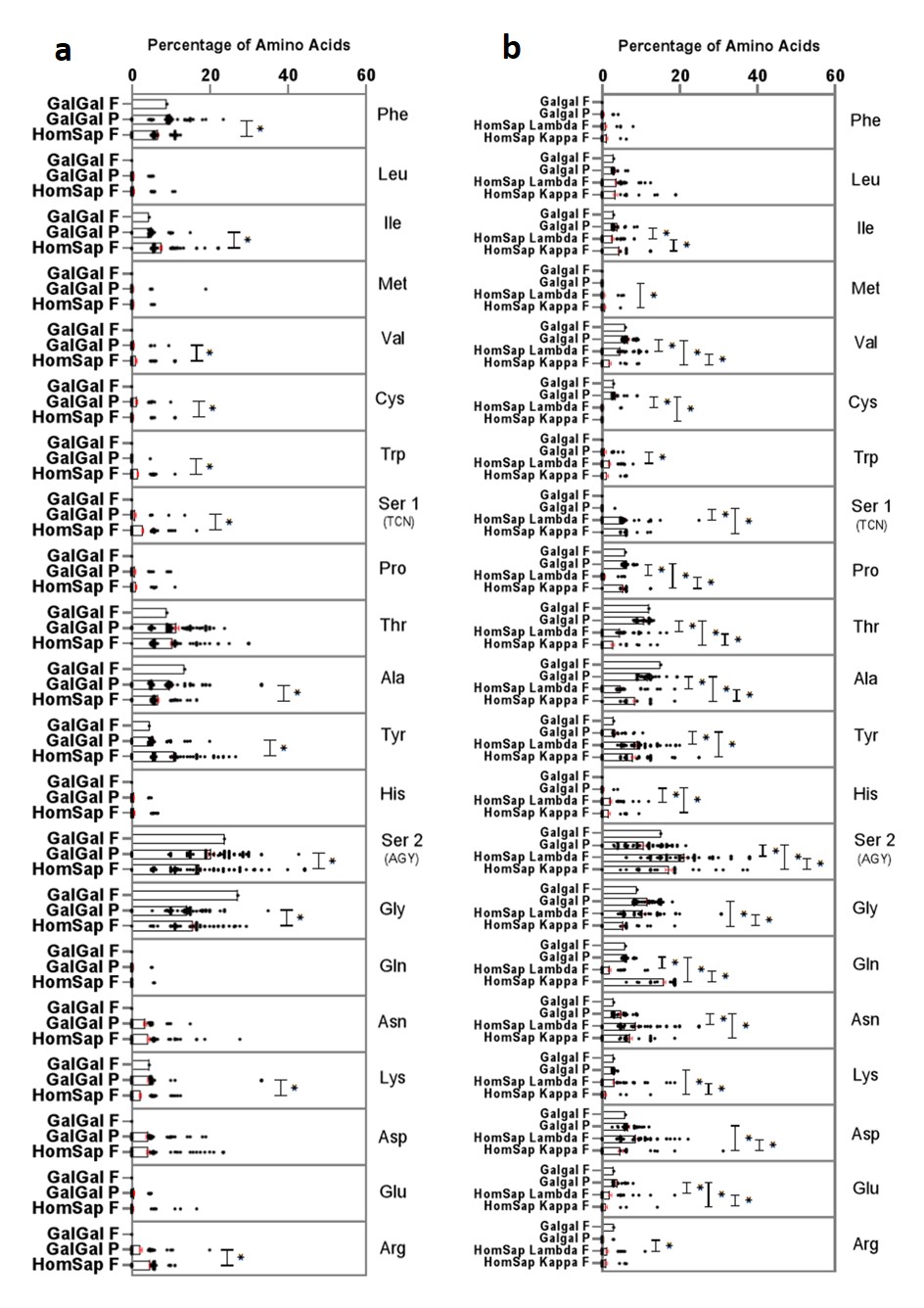

Supplement: Supplementary file 2 [file Image_2.jpeg]
