# Supplementary material for: Chickens, more than humans, focus the diversity of their immunoglobulin genes on the complementarity-determining region but utilise amino acids, indicative of a more cross-reactive antibody repertoire
Source: Front Immunol. 2022 Dec 8;13:837246. doi: 10.3389/fimmu.2022.837246 (PMC9772431; doi:10.3389/fimmu.2022.837246)
Supplement: Supplementary file 3 [file DataSheet_1.docx]

| Amino Acid | Hydrophobicity Score |
| --- | --- |
| Isoleucine | 4.92 |
| Valine | 4.04 |
| Leucine | 4.92 |
| Phenylalanine | 2.98 |
| Cysteine | 1.28 |
| Methionine | 2.35 |
| Alanine | 1.81 |
| Glycine | 0.94 |
| Threonine | -2.57 |
| Serine | -3.4 |
| Tryptophan | 2.33 |
| Tyrosine | -0.14 |
| Proline | 0 |
| Histidine | -4.66 |
| Glutamic Acid | -6.81 |
| Glutamine | -5.54 |
| Aspartic Acid | -8.72 |
| Asparagine | -6.64 |
| Lysine | -5.55 |
| Arginine | -14.92 |

Supplementary Table 1: Boman Index values for each amino acid (Boman. H. G 2003).

| Amino Acid | Hydrophobicity Score |
| --- | --- |
| Isoleucine | 4.5 |
| Valine | 4.2 |
| Leucine | 3.8 |
| Phenylalanine | 2.8 |
| Cysteine | 2.5 |
| Methionine | 1.9 |
| Alanine | 1.8 |
| Glycine | -0.4 |
| Threonine | -0.7 |
| Serine | -0.8 |
| Tryptophan | -0.9 |
| Tyrosine | -1.3 |
| Proline | -1.6 |
| Histidine | -3.2 |
| Glutamic Acid | -3.5 |
| Glutamine | -3.5 |
| Aspartic Acid | -3.5 |
| Aparagine | -3.5 |
| Lysine | -3.9 |
| Arginine | -4.5 |

Supplementary Table 2: Hydrophobicity Index values for each amino acid (Kyte. J, Doolittle. R. F. 1982).

| Second Amino Acid in Dipeptide | **L** | 13.3 | 20.26 | 1 | 1 | 1 | 1 | 1 | 1 | 20.26 | 1 | 1 | 1 | 1 | -7.49 | 1 | 1 | 1 | 1 | 1 | 1 |
| --- | --- | --- | --- | --- | --- | --- | --- | --- | --- | --- | --- | --- | --- | --- | --- | --- | --- | --- | --- | --- | --- |
|  | **A** | -14.03 | 1 | 13.34 | 1 | 24.68 | 1 | 1 | 1 | 1 | 1 | 1 | 20.26 | 1 | 1 | 1 | 1 | 1 | -7.49 | 1 | 1 |
|  | **G** | -9.37 | 1 | 1 | -9.37 | -7.49 | 1 | 1 | -14.03 | 1 | -7.49 | 1 | 1 | -7.49 | -7.49 | 1 | -7.49 | 1 | 13.34 | 1 | 1 |
|  | **S** | 1 | 1 | 44.94 | 1 | 1 | 1 | 44.94 | 1 | 1 | 44.94 | 20.26 | 20.26 | 1 | 1 | 20.26 | 1 | 20.26 | 1 | 1 | 1 |
|  | **V** | -7.49 | -6.54 | 1 | 1 | 1 | 1 | -6.54 | 1 | -7.49 | 1 | 1 | 20.26 | 1 | -7.49 | 1 | 1 | 1 | 1 | 1 | 1 |
|  | **E** | 1 | 1 | 1 | 1 | -6.54 | 1 | 20.26 | 1 | 44.94 | 1 | 1 | 18.38 | 20.26 | 1 | 33.6 | 1 | 20.26 | -6.54 | 1 | 1 |
|  | **K** | 1 | 1 | -6.64 | 24.68 | 1 | -14.03 | 1 | 24.68 | -7.49 | 1 | -7.49 | 1 | 1 | 1 | 1 | -1.88 | 1 | -7.49 | 1 | -7.49 |
|  | **T** | -14.03 | 33.6 | -1.88 | -6.54 | -7.49 | 1 | 1 | -7.49 | 1 | 1 | -14.03 | 1 | 1 | 1 | 1 | -7.49 | 1 | -7.49 | 1 | 1 |
|  | **P** | 1 | 20.26 | 44.94 | -1.88 | 13.34 | 20.26 | 20.26 | -1.88 | -1.88 | 20.26 | 1 | 20.26 | 1 | -6.54 | 20.26 | 20.26 | 44.94 | 1 | 20.26 | 20.26 |
|  | **D** | 1 | 20.26 | 1 | 1 | 24.68 | 13.34 | 20.26 | 1 | 1 | 1 | 1 | -6.54 | 1 | 1 | 20.26 | -14.03 | 1 | 1 | -7.49 | 1 |
|  | **R** | 1 | 1 | -6.54 | 1 | -15.91 | 1 | 1 | 1 | 1 | 58.28 | -6.54 | -6.54 | 1 | 33.6 | 1 | 1 | 20.26 | 1 | 1 | 20.26 |
|  | **I** | 1 | 1 | 1 | 44.94 | 1 | 1 | 1 | 44.94 | 1 | 1 | 1 | 1 | 1 | -7.49 | 20.26 | 1 | 1 | -7.49 | 1 | 1 |
|  | **N** | 13.34 | 1 | 1 | 24.68 | 1 | 1 | 1 | 1 | 1 | 13.34 | 1 | 1 | -14.03 | 1 | 1 | 1 | 1 | -7.49 | 1 | 1 |
|  | **Q** | 1 | -6.54 | -6.54 | 1 | 1 | 1 | 20.26 | -6.54 | 1 | 20.26 | 1 | 20.26 | -6.54 | 24.68 | 20.26 | 1 | 20.26 | 1 | 1 | 33.6 |
|  | **F** | 1 | 1 | 1 | -9.37 | 1 | 1 | -6.54 | -14.03 | 1 | 1 | -6.54 | 20.26 | 13.34 | 1 | 1 | 1 | 1 | 1 | 1 | 1 |
|  | **Y** | 1 | 1 | 24.68 | 44.94 | 13.34 | 33.6 | -6.54 | 1 | 1 | -6.54 | 1 | 1 | 1 | 1 | 1 | -6.54 | 1 | -7.49 | 1 | 1 |
|  | **H** | 24.68 | 33.6 | 58.28 | 1 | 13.34 | 1 | 1 | 1 | 13.34 | 20.26 | 1 | 1 | 1 | 1 | -6.54 | 1 | 1 | 1 | -7.49 | 1 |
|  | **M** | 24.68 | 33.6 | -1.88 | 1 | 44.94 | 1 | 1 | 1 | 1 | 1 | 1 | -6.54 | 1 | 33.6 | 1 | 1 | 1 | 1 | 1 | 1 |
|  | **C** | 1 | 1 | 1 | 1 | 1 | 1 | -6.54 | -1.88 | 1 | 1 | 1 | -6.54 | 1 | 1 | 44.94 | 1 | 33.6 | 1 | 44.94 | 1 |
|  | **W** | 1 | 24.68 | 1 | -1.88 | -9.37 | 1 | 1 | -9.37 | 1 | 58.28 | 1 | -1.88 | -14 | 1 | -14 | 1 | 1 | 13.34 | 1 | 24.688 |
|  | | **W** | **C** | **M** | **H** | **Y** | **F** | **Q** | **N** | **I** | **R** | **D** | **P** | **T** | **K** | **E** | **V** | **S** | **G** | **A** | **L** |
|  |  | First Amino Acid in Dipeptide | | | | | | | | | | | | | | | | | | | |

Supplementary Table 3: Instability Index values for each dipeptide combination (Guruprasad. K, et al 1990).

Supplementary Figure 1: Comparison of amino acid usage across all three FWR regions of the variable germline genes of both chickens and humans, displaying amino acid usage in **A**) the immunoglobulin heavy chain, **B**) immunoglobulin light chain. Amino acids: Phenylalanine (Phe), Leucine (Leu), Isoleucine (Ile), Methionine (Met), Valine (Val), Cysteine (Cys), Tryptophan (Trp), Serine 1 (Ser 1 – TCN), Proline (Pro), Threonine (Thr), Alanine (Ala), Tyrosine (Tyr), Histidine (His), Serine 2 (Ser 2- AGY), Glycine (Gly), Glutamine (Gln), Asparagine (Asn), Lysine (Lys), Aspartic Acid (Asp), Glutamic Acid (Glu), Arginine (Arg). Datasets were statistically analysed using a One-Way ANOVA. P values: ns = P > 0.05, * = P ≤ 0.05.

Supplementary Figure 2: Comparison of amino acid usage across all three CDR regions of the variable germline genes of both chickens and humans, displaying amino acid usage in **A**) the immunoglobulin heavy chain, **B**) immunoglobulin light chain. Amino acids: Phenylalanine (Phe), Leucine (Leu), Isoleucine (Ile), Methionine (Met), Valine (Val), Cysteine (Cys), Tryptophan (Trp), Serine 1 (Ser 1 – TCN), Proline (Pro), Threonine (Thr), Alanine (Ala), Tyrosine (Tyr), Histidine (His), Serine 2 (Ser 2- AGY), Glycine (Gly), Glutamine (Gln), Asparagine (Asn), Lysine (Lys), Aspartic Acid (Asp), Glutamic Acid (Glu), Arginine (Arg). Datasets were statistically analysed using a One-Way ANOVA. P values: ns = P > 0.05, * = P ≤ 0.05.
